# Study on the role of Shenfu injection in mediating ferroptosis through the Akt/GSK-3β/Nrf2 pathway in yang-deficient chronic heart failure

**DOI:** 10.55730/1300-0152.2777

**Published:** 2025-06-11

**Authors:** Xiaojie CHEN, Jiayun GUO, Bing LIN, Huamin WANG, Ziyu LU, Bayi LIU

**Affiliations:** 1 Department of Critical Care Medicine, The Tenth Clinical Medical College of Guangzhou University of Traditional Chinese Medicine, Zhongshan City, Guangdong Province, China; 2 Department of Critical Care Medicine, Zhongshan Hospital of Traditional Chinese Mediine Affiliated to Guangzhou University of Traditional Chinese Medicine, Zhongshan City, Guangdong Province, China

**Keywords:** Shenfu injection, yang-deficient chronic heart failure, Akt/GSK-3β/Nrf2 pathway, ferroptosis

## Abstract

**Background/aim:**

The present study investigates the role of Shenfu injection in the treatment of yang-deficient chronic heart failure (CHF).

**Materials and methods:**

Sprague-Dawley (SD) rats were modeled for yang-deficient CHF by abdominal aortic coarctation. Echocardiography was performed to detect changes in cardiac function, and serum N-terminal B-type natriuretic peptide proteins (NT-proBNP), cardiac troponin I (cTnI), and ferroptosis-related factors were measured using ELISA kits. Pathological changes in cardiac tissues were observed through hematoxylin-eosin (HE) and Masson’ trichrome staining, cardiomyocyte apoptosis was measured by TUNEL staining, and reactive oxygen species (ROS) production was determined through dihydroethidium (DHE) staining. The expression of nuclear factor E2-related factor 2 (Nrf2), cyclooxygenase 2 (Ptgs2), glutathione peroxidase 4 (GPX4), solute carrier family 3 member 2 (SLC3A2), solute carrier family 7 member 11 (SLC7A11), and acyl-CoA synthetase long-chain family member 4 (ACSL4) in cardiac tissues were analyzed through RT-qPCR. Phosphorylated Akt (p-Akt), phosphorylated GSK-3β (p-GSK-3β), and Nrf2 expression in tissues were tested through immunohistochemistry. The protein expression of the Akt/GSK-3β/Nrf2 pathway was detected by Western blot. The Akt/GSK-3β/Nrf2 pathway inhibitor LY294002 was applied to the rats administrated with Shenfu injection.

**Results:**

Shenfu injection decreased the left ventricular end-diastolic diameter and left ventricular end-systole diameter and increased the left ventricular ejection fraction and left ventricular fractional shortening in rats with CHF. The treatment reduced NT-proBNP and cTnI levels, while improving pathological damage in the cardiac tissue. The treatment was also noted to decrease serum MDA, ACSL4, and Fe^2+^ and increase GSH, GPX4, SOD, and SLC3A2 in the sample; increase GPX4,SLC7A11 and SLC3A2 mRNA in cardiac tissues, and decrease Ptgs2 and ACSL4 mRNA. Shenfu injection was also noted to activate the Akt/GSK-3β/Nrf2 signaling pathway, while LY294002 weakened the therapeutic effect of the treatment on cardiac tissue damage.

**Conclusion:**

Shenfu injection activates the Akt/GSK-3β/Nrf2 pathway to prevent myocardial injury and ferroptosis in yang-deficient CHF.

## 1. Introduction

Chronic heart failure (CHF) occurs at the end-stage of heart disease and is the primary cause of lost wages and death in people over the age of 70 years (Yang et al., 2016; Andries et al., 2019). Studies of the use of Chinese medicine injections for the treatment of heart failure (HF) have been increasing in recent years owing to their reported ability to improve symptoms and the efficacy of Western drug therapies, while effectively reducing their side effects or adverse reactions (Chen et al., 2022). In traditional Chinese medicine, the symptoms of HF are generally attributed to qi and yang deficiencies while the main diagnostic typology is heart and kidney yang deficiency (Cheng et al., 2025; Dodson et al., 2019). The main symptoms of HF include palpitations, shortness of breath, fatigue, cold extremities, swelling, abdominal distension, diarrhea, dull complexion, white tongue with thin moss, weak or tense pulse, astringency, and junction generation (Fang et al., 2020). In TCM theory, the pathogenesis of CHF involves yang deficiency in the heart and kidneys, which is a key mechanism indicative of a more severe pathologic phenotype. These factors lead to fatigue and disrupt organ balance, resulting in poor blood and fluid flows that increase the burden on the heart and promote myocardial fibrosis (Gao et al., 2021).

The injectable form of Shenfu is derived from Shenfu decoction, a formula in TCM that works on the principle of warming yang made from extracts of *Radix Ginseng Rubra* and *Aconitum carmichaeli Debx*. After being granted marketing authorization in 1987, it has been widely used in clinical settings for the treatment of shock, cardiopulmonary resuscitation, and HF (Guo et al., 2021). Furthermore, Shenfu injection has been shown to improve cardiac function and reduce inflammation and fibrosis associated with in CHF rats (Huang et al., 2022), and its clinical efficacy and safety have been confirmed as a CHF treatment (Jia et al., 2020). Shenfu injection has been shown to improve left ventricular ejection fraction (LVEF) and quality of life in CHF patients (Kerins and Ooi, 2018), and when combined with tachycardia, it has been shown to significantly improve cardiac function and reduce the incidence of adverse effects in patients with CHF with accompanying coronary artery disease (Li et al., 2014). Despite its wide clinical application, however, the mechanism of action of Shenfu injection for the treatment of CHF is not clear.

Ferroptosis is a new form of cell death that is caused by excessive intracellular lipid peroxidation, and has received widespread attention within the medical field. Ferroptosis causes damage to cardiomyocytes, and the subsequent necrosis of these cells can evolve into tissue scarring, affecting cardiac function and leading ultimately to HF (Liu et al., 2018; Liu et al., 2020; Liu et al., 2021; Mei et al., 2022; Palacios Ordonez and Garan, 2022). Puerarin can partially inhibit the ferroptosis process in cardiomyocytes by inducing the production of glutathione peroxidase 4 (GPX4) and decreasing the production of ROS (Sheng et al., 2021). As a ferroptosis inducer, erastin increases ROS levels and decreases cardiomyocyte activity, leading subsequently to HF (Špinar et al., 2018).

The relationship between the PI3K/Akt/GSK3 pathway and HF has been studied and confirmed (Tian et al., 2022). The activation of nuclear factor erythroid 2–related factor 2 (Nrf2) has a palliative effect on HF and is a downstream target of the PI3K/AKT pathway (Vashi and Patel, 2021; Wang et al., 2022a). Nrf2 regulates ferritin, thereby maintaining cellular iron homeostasis (Wang et al., 2017). Deficiencies in Nrf2 exacerbate decompensated cardiac remodeling, characterized by increased myocardial apoptosis, fibrosis, oxidative stress, and cardiac hypertrophy (Wang et al., 2019). Nrf2 regulates intracellular iron homeostasis by controlling the expression of downstream genes. Promoting Nrf2 nuclear translocation can upregulate the expression of the ferroptosis-related proteins, thereby protecting the cells from ferroptosis (Wang et al., 2022b).

The present study investigates whether Shenfu injection ameliorates myocardial injury in rats with yang-deficient CHF by mediating ferroptosis through the Akt/GSK-3β/Nrf2 pathway, thereby providing a theoretical basis for its clinical optimization and application.

## 2. Materials and methods

### 2.1. Animals and groups

Included in the study were 70 male Sprague-Dawley (SD) rats aged 2–3 months and weighing 230–250 g, acquired from the Institute of Laboratory Animals Science (Beijing, China). All rats were housed for a week in a specific pathogen-free (SPF)-grade animal house kept at a temperature of 25 ± 2 °C and a relative humidity of 30~60%, in alternating light and dark (12 h/12 h), and with sufficient water and food. The experimental protocol was approved by the Ethics Review Committee of Zhongshan Affiliated Hospital, Guangzhou University of Chinese Medicine. The rats were divided into a control group, a model group, a Shenfu injection low-dose group, a Shenfu injection medium-dose group, a Shenfu injection high-dose group, a Shenfu injection high-dose group + LY294002 group, and positive control group, with 10 rats assigned to each group.

### 2.2. CHF modeling

The CHF rat model was established through abdominal aortic coarctation, i.e., the rat was anesthetized and fixed on the operating table on its back, the chest and abdomen were shaved and disinfected, and the abdominal cavity was opened layer by layer to isolate the abdominal aorta. A No. 6 needle was inserted in the abdominal aorta, a 4-0 silk thread was passed through the segment of the abdominal aorta and ligated together with the No. 6 needle and the abdominal aorta, and the needle was rapidly withdrawn after ligation. The abdominal aorta was constricted to 60%–70% of its original diameter, sutured, and treated with standard anti-infective therapy. After 1 week of normal feeding, the yang deficiency modeling was initiated, for which the appropriate rats were kept at temperatures ranging from −2 °C to −4 °C for 2 h a day for 4 weeks (Wang et al., 2021). Echocardiography (ECG) was used to detect LVEF in rats, with LVEF ≤ 45% indicating a successful achievement of a CHF rat model. At the end of the experiment, six surviving rats were randomly selected, and after testing left ventricular function, cardiac tissues were collected and fixed in 4% paraformaldehyde. The criteria used to indicate kidney yang deficiency included decreased body temperature, huddling behavior, raised fur, sluggish movement, lethargy, a cold and dull tail, reduced dorsal temperature, and swelling on the back and feet. Pathologically, focal myocardial degeneration, swelling, and fibroplasia, along with hypertrophy and disordered arrangement of cardiomyocytes, obvious interstitial fibrosis, and ventricular remodeling, all indicated successful model replication.

For the establishment of the Shenfu injection groups, 4, 8, and 16 mL/kg of Shenfu injection were intraperitoneally injected, respectively, while for the establishment of the positive control group, 13.5 mg/kg of captopril was administered by gavage. The control group and the model group were administered an equal amount of 0.9% sodium chloride solution once a day for 4 weeks. The high-dose Shenfu injection group was given weekly tail vein injections of LY294002 (0.5 mg/kg) on the premise of 16 ml/kg Shenfu injection per day. On the 29th day, the rats were anesthetized with 10% chloral hydrate by intraperitoneal injection, and blood was collected from the abdominal aorta, left to stand for 30 min, and centrifuged (3000 rpm, 15 min), and the separated serum was stored at −80 °C. The skin was wiped with 75% alcohol, a large V shape was cut into the abdomen to expose the abdominal aorta, and the heart was extracted, rinsed with 4 °C saline, dried, and weighed on electronic scales to calculate the heart quality index. The left ventricle was placed in 4% paraformaldehyde and stored at room temperature for pathological testing, while the other cardiac tissue was frozen in liquid nitrogen at −80 °C.

### 2.3. ECG

At the end of drug administration, the rats were anesthetized through the intraperitoneal injection of 1% pentobarbital sodium (50 mg/kg) and fixed in the supine position. The left ventricular end-diastolic diameter (LVEDD), left ventricular end-systolic diameter (LVESD), LVEF, and left ventricular fractional shortening (LVFS) were detected by ECG, obtained from short-axis M-mode scans at the level of the central ventricle.

### 2.4. HE staining

The left ventricular cardiac tissue was fixed in 4% paraformaldehyde embedded in paraffin, and sectioned into 4-μm tissue slices. For HE staining, the tissue sections were deparaffinized with xylene, stained with hematoxylin solution for 5 min, rinsed with running water until the sections returned to blue, stained with eosin solution for 3 min, dehydrated with gradient ethanol, cleared with xylene, and sealed with neutral gum, following the instructions of the HE staining kit manufacturer (Servicebio, Wuhan, China). Pathological changes in the cardiac tissues were examined under optical microscope (Mshot MF53, Guangzhou, China).

### 2.5. Masson staining

The left ventricle was fixed in 4% paraformaldehyde and sectioned into 4 μm slices, deparaffinized, stained sequentially with potassium dichromate, hematoxylin, poncean red, phosphomolybdic acid, and aniline blue, differentiated for 3 min in 1% acetic acid solution, dehydrated in absolute alcohol, cleared with xylene, and sealed with neutral gum, following the instructions of Masson staining kit manufacturer (Solarbio, Beijing, China). Myocardial fibrosis was examined under a light microscope.

### 2.6. TUNEL staining

Myocardial paraffin sections were dewaxed and dehydrated, treated with 3% H_2_O_2_, and digested with proteinase K for 20 min. The TUNEL (Servicebio) reaction solution was added and incubated for 1 h at 37 °C in a humidified chamber, followed by incubation with the POD converter for 30 min at 37 °C and staining with a DAB solution (ZSGB-BIO, Beijing, China). The sections were then sealed and examined, and cells with brown particles in the nucleus were considered apoptotic. The apoptosis rate was calculated as: (apoptotic cells/total number of cells) × 100%.

### 2.7. ROS detection

Frozen cardiac tissue was cut into sections of approximately 4 μm thickness, placed on slides, incubated with 10 μM DHE (Sigma-Aldrich) at 37 °C for 30 min, blocked with an antifluorescence quencher, and visualized under fluorescence microscope (535 nm).

### 2.8. ELISA and Fe^2+^ detection

For serum myocardial function-related factors, including NT-proBNP and cTnI, were measured using the corresponding assay kits (respectively, RX302959R, Ruixin Biotech, Quanzhou, China; and CB11507-Ra, Coibio-Bio, Shanghai, China). For the detection of serum ferroptosis-related markers, the appropriate assay kits were used to detect glutathione (GSH, CB10393-Ra, Coibio-Bio), malondialdehyde (MDA, CB10238-Ra, Coibio-Bio), GPX4 (RX300558R, Ruixin Biotech), superoxide dismutase (SOD, CB10258-Ra, Coibio-Bio), acyl-CoA synthetase long-chain family member 4 (ACSL4, CB12906-Ra, Coibio-Bio), solute carrier family 3 member 2 (SLC3A2, F21604-A, SHFKSC, Shanghai, China) and Fe^2+^ (E-BC-K772-M2, Elabscience). To ensure the accuracy and reliability of the experimental results, each assay batch included a negative control (in which serum samples were replaced with PBS), a positive control (using the standards provided by the kit), and a blank control (containing only the test reagents), with at least two duplicate wells for each control. Serum samples were also prepared for the high, medium, and low concentrations as an internal quantitative quality control, and were tested simultaneously with the samples, with recoveries of between 80% and 120% and coefficients of variation < 15%. To ensure the reliability of the experimental data, the samples were routinely subjected to third-party quality control product comparisons.

### 2.9. RT-qPCR

Total RNA was extracted from the cardiac tissues using TRIzol reagent (Invitrogen) and reverse transcribed into cDNA with the Goldenstar RT6 cDNA Synthesis Kit Ver. 2 (Tsingke Biotechnology, Beijing, China). RT-qPCR was performed using a 2×T5 Fast qPCR Mix kit (SYBR Green I, Tsingke Biotechnology) on a BIO-RAD iQ5 Real-Time Fluorescent Quantitative PCR System. At the end of the amplification process, the relative expression of the genes was calculated using the 2^−ΔΔCt^ method with GAPDH used as the internal reference. The primer sequences are presented in Table.

### 2.10. Immunohistochemistry

Tissue sections were fixed in formalin and embedded in paraffin before sectioning (4 μm). The sections were incubated overnight with primary antibodies, including anti-p-AKT (#4060, Cell Signaling Technology), anti-p-GSK-3β (#9323, Cell Signaling Technology), and anti-Nrf2 (#DF8023, affinity). The sections were washed repeatedly for 30 min prior to incubation using a goat anti-rabbit secondary antibody (#511203, ZEN-BIOSCIENCE, Chengdu, China). The sections were then stained with DAPI (Beyotime, China), re-stained with hematoxylin (Servicebio), dehydrated in absolute alcohol, cleared in xylene, mounted with neutral gum, and examined under an Mshot MF53 inverted microscope (Microshot, Guangzhou, China).

### 2.11. Western blot

Total proteins were extracted from tissue sections using RIPA lysis buffer (Beyotime) and protein concentrations were quantified using a BCA kit (Beyotime). Equal amounts of protein were separated using 10–15% SDS-PAGE, and the samples were transferred to polyvinylidene difluoride membranes (EMD Millipore, MA, USA), closed with 5% skimmed milk powder for 1 h at room temperature, and incubated overnight at 4 °C with anti-AKT (#9272, Cell Signaling Technology), anti-p-AKT (#4060, Cell Signaling Technology), anti-GSK-3β (#12456, Cell Signaling Technology), anti-p-GSK-3β (#9323, Cell Signaling Technology), and anti-Nrf2 (#DF8023, affinity). The membranes were then incubated with horseradish peroxidase-coupled goat anti-rabbit secondary antibody (#511203, ZEN-BIOSCIENCE) for 1 h at room temperature and measured using an ECL kit (EMD Millipore).

## 3. Statistical analysis

Statistical analyses and graphing were performed using GraphPad Prism 10.0 software (GraphPad Software, La Jolla, CA). All data in the graphs are presented as mean ± standard deviation. Statistical comparisons of two groups of data were performed using an unpaired two-tailed Student’s t-test, while comparisons among multiple groups were performed using one-way ANOVA. A p-value of 0.05 was considered statistically significant.

## 4. Results

### 4.1. Shenfu injection improves cardiac tissue damage in rats with yang-deficient CHF

**Figure 1:**
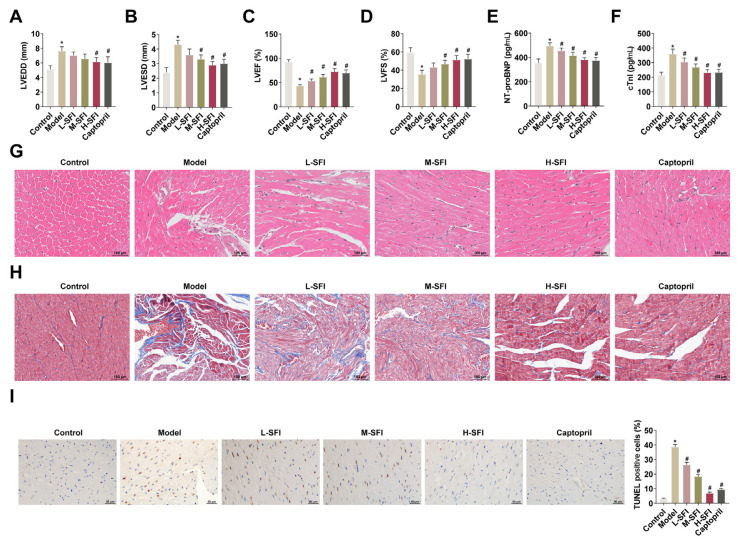
Shenfu injection improves cardiac tissue damage in rats with yang-deficient CHF. A–D: ECG showing the changes of LVEDD, LVESD, LVEF, and LVFS; E, F: ELISA kit detecting NT-proBNP and cTnI; G, H: HE and Masson staining, revealing pathological changes in the cardiac tissues in each group of rats; I: TUNEL staining revealing cardiac tissue apoptosis in each group of rats. Data were expressed as mean ± SD. *p < 0.05 compared with control group, and #p < 0.05 compared with the model group.

After modeling, the LVEDD, LVESD, LVEF, and LVFS of the rats were measured ECG to confirm the success of modeling. LVEDD and LVESD values were higher and LVEF and LVFS values were lower in the model group, and LVEF was < 45%, which indicated that cardiac function was impaired and that the CHF modeling had been successful (Figure 1, A–D). It was noted that Shenfu injection restored cardiac function in a dose-dependent manner in CHF rats. The elevated serum NT-proBNP and cTnI levels recorded in the CHF rats were reduced by Shenfu injection (Figures 1 E and 1F). Next, pathological changes of rat cardiac tissues were observed using HE and Masson staining. HE staining revealed that cardiomyocytes in the model group were thickened, disordered in arrangement, and showed different degrees of swelling and degeneration, whereas no such pathological changes were observed in the control group. Mild hypertrophy was observed in the Shenfu injection treatment groups and the positive control group, but no swelling or degeneration seen (Figure 1G). Masson staining revealed obvious myocardial fibrosis and hypertrophy in the model group, and Shenfu injection ameliorated the pathological damage of cardiac tissues in a dose-dependent manner, with the high-dose Shenfu injection demonstrating the most pronounced improvement effect (Figure 1H). The results of HE and Masson staining indicated the successful replication of the rat kidney yang deficiency model. Finally, TUNEL staining showed that CHF induced extensive apoptosis in cardiac tissue, which was reduced following Shenfu injection in a dose-dependent manner (Figure 1I).

### 4.2. Shenfu injection reduces ferroptosis in cardiac tissues of rats with yang-deficient CHF

**Figure 2:**
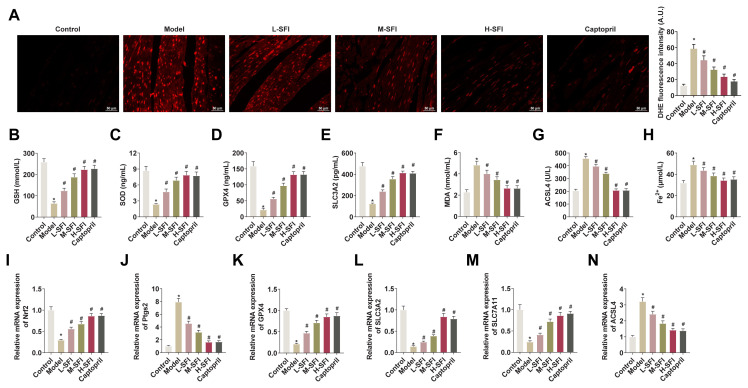
Shenfu injection reduces ferroptosis in cardiac tissues of rats with yang-deficient CHF. A: DHE probe detecting ROS in the cardiac tissues of rats in each group; B–G: ELISA detecting GSH, SOD, GPX4, SLC3A2, MDA, and ACSL4 in serum of rats in each group; H: Fe^2+^ kit detecting Fe2+ in the serum of rats in each group; I: RT-qPCR detecting Nrf2 in the cardiac tissues of rats in each group; J–N: RT-qPCR detecting Ptgs2, GPX4, SLC3A2, SLC7A11, and ACSL4 in the cardiac tissues of rats in each group. Data were expressed as mean ± SD. *p < 0.05 compared with control group, and #p < 0.05 compared with the model group.

Shenfu injection decreased ROS levels in cardiac tissues (Figure 2A). ELISA results showed that serum GSH, SOD, GPX4, and SLC3A2 were decreased, and MDA, and ACSL4 were increased in CHF rats, and that Shenfu injection upregulated serum GSH, SOD, GPX4, and SLC3A2 levels and decreased MDA and ACSL4 levels in a dose-dependent manner (Figures 2B–2G). The F^e2+^ concentration in the serum of rats in the model group was increased, suggesting that CHF induces ferroptosis, but was decreased by Shenfu injection in a dose-dependent manner (Figure 2H). Nrf2 plays an important role in ferroptosis resistance. In RT-qPCR experiments, a marked reduction in Nrf2 expression was observed in rats with CHF. However, administration of Shenfu injection mitigated this reduction in Nrf2 levels (Figure 2I). The RT-qPCR experiments also revealed a decrease in ferroptosis factors GPX4, SLC3A2, and SLC7A11 in the model group, and elevated Ptgs2 and ACSL4. Subsequent Shenfu injection led to an upregulation of GPX4, SLC3A2, and SLC7A11 and a reduction in Ptgs2 and ACSL4 (Figures 2J–2N).

### 4.3. Shenfu injection regulates the activation of the Akt/GSK-3β/Nrf2 pathway

**Figure 3:**
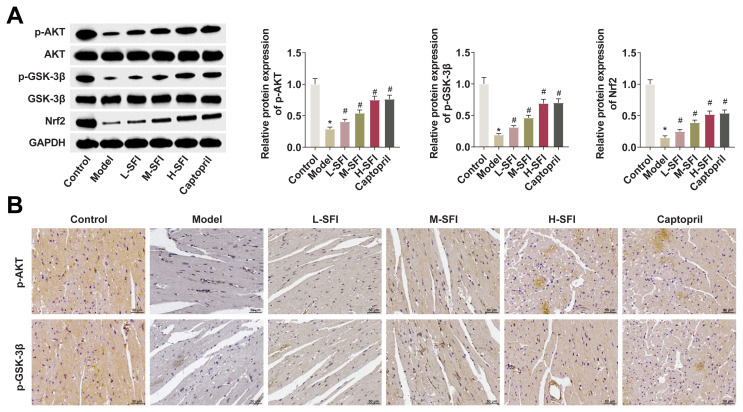
Shenfu injection regulates the activation of Akt/GSK-3β/Nrf2 pathway. A: Western blot detecting p-AKT, p-GSK-3β, and Nrf2 in the cardiac tissues of rats in each group; B: Immunohistochemistry detecting p-AKT, p-GSK-3β, and Nrf2 in the cardiac tissues of rats in each group. Data were expressed as mean ± SD. *p < 0.05 compared with control group, and #p < 0.05 compared with the model group.

To investigate the specific mechanism behind the upregulation of Nrf2 activity following Shenfu injection, the activation of the Akt/GSK-3β pathway was detected by Western blot. The reduced p-levels of AKT, p-GSK-3β, and Nrf2 observed in the cardiac tissues of the rats in the model group were significantly restored following Shenfu injection (Figure 3A). Similar results were obtained immunohistochemically, revealing the decreased expression of p-AKT, p-GSK-3β, and Nrf2 to be partially resolved following Shenfu injection (Figure 3B).

### 4.4. Akt/GSK-3β/Nrf2 pathway inhibitor reverses the therapeutic effects of Shenfu injection on cardiac tissue damage in yang-deficient CHF rats

**Figure 4:**
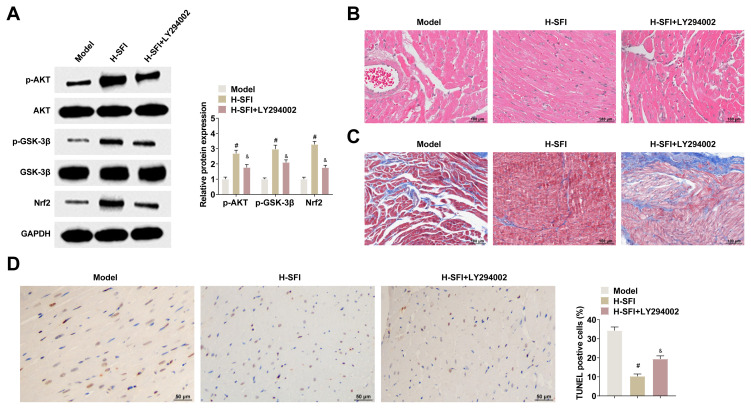
Akt/GSK-3β/Nrf2 pathway inhibitor reverses the therapeutic effects of Shenfu injection on cardiac tissue damage in yang-deficient CHF rats. A: Western blot detecting p-AKT, p-GSK-3β, and Nrf2 in the cardiac tissue of rats; B, C: HE staining with Masson revealing the pathology of cardiac tissues of rats in each group; D: TUNEL detecting cardiac tissue apoptosis in rats in each group. Data were expressed as mean ± SD. *p < 0.05 compared with control group, and ^#^p < 0.05 compared with the high-dose Shenfu injection group, ^&^p < 0.05.

To further elucidate the regulation of Akt/GSK-3β/Nrf2 in yang-deficient CHF rats, appropriate rats were treated with high-dose Shenfu injection, with or without the LY294002 pathway inhibitor. The inhibitory effect of LY294002 was verified by Western blot. LY294002 was noted to significantly reduce p-AKT, p-GSK-3β, and Nrf2 in rats treated with high-dose Shenfu injection (Figure 4A). HE and Masson staining revealed that LY294002 weakened the therapeutic effect of high-dose Shenfu injection in rats with cardiac tissue injury (Figures 4B and 4C). The TUNEL results revealed that the coadministration of LY294002 increased apoptosis in rat cardiac tissues when compared with high-dose Shenfu injection (Figure 4, D).

### 4.5. Shenfu injections regulate ferroptosis in cardiac tissues of yang-deficient CHF rats via the Akt/GSK-3β/Nrf2 pathway

**Figure 5:**
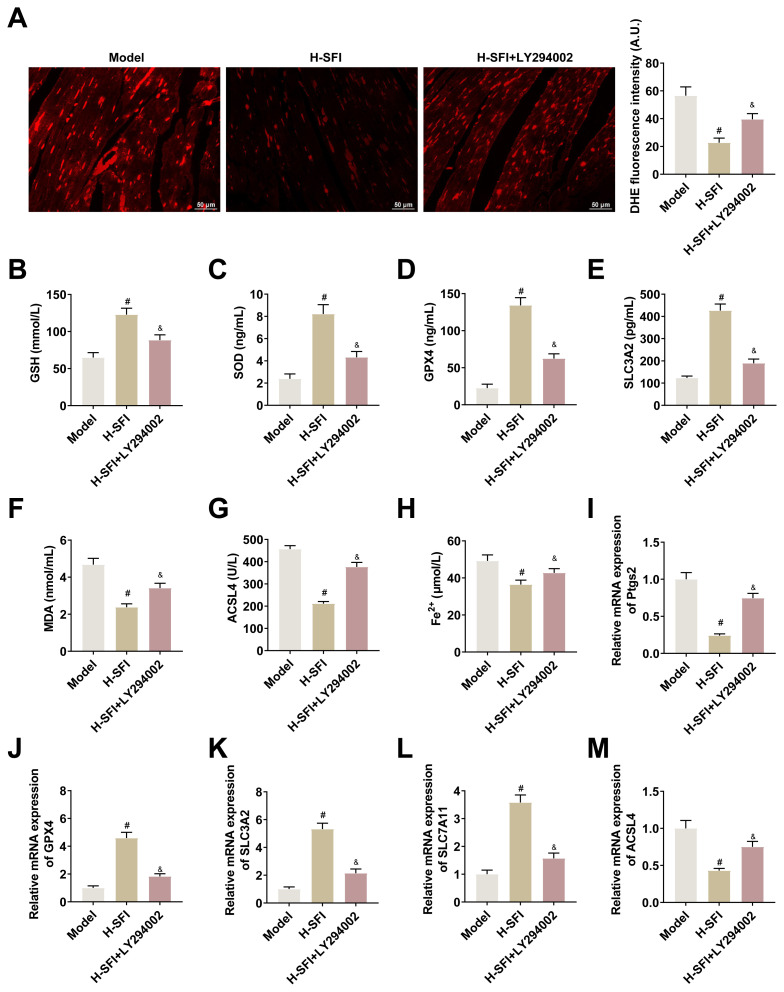
Shenfu injections regulate ferroptosis in cardiac tissues of yang-deficient CHF rats via Akt/GSK-3β/Nrf2 pathway. A: DHE probe detecting ROS in the cardiac tissues of rats in each group; B–G: ELISA detecting GSH, SOD, GPX4, SLC3A2, MDA, and ACSL4 in serum of rats in each group; H: F^e2+^ kit detecting Fe2+ in serum of rats in each group; I: RT-qPCR detecting Nrf2 in the cardiac tissues of rats in each group; J–M: RT-qPCR detecting Ptgs2, GPX4, SLC3A2, SLC7A11, and ACSL4 in the cardiac tissues of rats in each group. Data are expressed as mean ± SD. *p < 0.05 compared with control group, and ^#^p < 0.05 compared with the high-dose Shenfu injection group, ^&^p < 0.05.

In an investigation of the effect of Akt/GSK-3β/Nrf2 pathway inhibitor LY294002 on ferroptosis in the cardiac tissues of yang-deficient CHF rats, LY294002 was noted to weaken the effect of high-dose Shenfu injection on the reduction of ROS (Figure 5A). Moreover, LY294002 was noted to weaken the high-dose Shenfu injection-induced upregulation of GSH, SOD, GPX4, and SLC3A2 levels and the reduction of MDA and ACSL4 levels (Figures 5B–5G). Furthermore, LY294002 was noted to increase Fe^2+^ levels, reversing the reductions achieved by high-dose Shenfu injection on Fe^2+^ concentration (Figure 5H). The RT-qPCR analysis also revealed that LY294002 weakened the upregulation of GPX4, SLC3A2, and SLC7A11 and the reduction of Ptgs2 and ACSL4 achieved through high-dose Shenfu injection in rat cardiac tissues (Figure 5I–5M).

## 5. Discussion

CHF is the final event in most cardiovascular diseases and is the leading cause of death (Wu et al., 2021). According to Traditional Chinese Medicine, yang deficiency and blood stasis are the main pathological changes in HF (Xu et al., 2024). At present, Western medicine relies mainly on conservative treatment, including angiotensin receptor blockers and β-blockers; however, the side effects associated with the long-term administration of drugs can lead to reduced patient compliance with treatment and failure to achieve the expected therapeutic effects (Yan et al., 2019). The clinical research concerning the application of modern Chinese medicine in the treatment of chronic heart failure (CHF) has seen significant advancements (Yu et al., 2019; Yang et al., 2022). Traditional Chinese Medicine (TCM) has garnered extensive experience in managing CHF, particularly in instances characterized by heart and kidney yang deficiency. Traditional Chinese herbal formulas such as Zhenwu, Bushen Huoxue, and Shenfu decoctions are commonly used to warm and tonify kidney-yang, and are often supplemented with combinations of medicines that invigorate blood circulation and eliminate blood stasis in order to improve symptoms and quality of life in patients with HF (Yu et al., 2021). In Shenfu injection, *Radix Ginseng Rubra* is believed to tonify qi, normalize blood pressure, tonify the spleen, benefit the lungs, and calm the mind, while *Aconitum carmichaelii Debx.* has the effects of restoring and tonifying yang, dispersing cold, and relieving pain. When given together, the two medicines are understood to benefit the qi and warm the yang. Modern pharmacological studies have shown that *Ginseng saponin*—the active ingredient in Shenfu injection—can expand the coronary artery, reduce myocardial oxygen consumption and peripheral vascular resistance. It also strengthens the repair of cardiomyocytes, improves the excitability of β-receptors of cardiomyocytes, aids in the retention of the positive inotropy of cardiomyocytes, reduces mitochondrial injury, and protects cardiomyocytes and the function of endothelial cells in the capillaries (Zhang et al., 2019; Zhao et al., 2022). In the present study, Shenfu injection was shown to produce favorable therapeutic effects in yang-deficient CHF rats, in which LVEDD and LVESD were elevated, LVEF and LVFS were reduced, and NT-proBNP and cTnI were significantly increased. The administration of Shenfu injection induced dose-dependent changes in these parameters, with the highest dose having the best therapeutic effect, comparable with that of the positive control. Shenfu injection was also noted to attenuate cardiac tissue damage in yang-deficient CHF rats in a dose-dependent manner. Together, these results confirm the favorable effects of Shenfu injection in the treatment of yang-deficient CHF.

Multiple modes of cell death occur during the development of HF, forming a complex regulatory network. The inhibition of ferroptosis—a new mode of programmed cell death that differs from apoptosis, autophagy, and pyroptosis—can reduce cardiomyocyte hypertrophy, improve ventricular dilatation, inhibit myocardial inflammatory response, and enhance myocardial diastolic function. The results of the vigorous efforts to develop Chinese medicine-related preparations for the treatment of myocardial injury after CHF based on ferroptosis have been promising. In the present study, Shenfu injection removed excess ROS and Fe^2+^ from the cardiac tissues of yang-deficient CHF rats, decreased MDA and ACSL4, and increased SOD, GSH, GPX4, SLC3A2, and SLC7A11, while also increasing Nrf2 and decreasing Ptgs2 expression.

Nrf2 is a key nuclear transcription factor that alleviates oxidative stress by regulating both the expression of a range of signaling proteins and enzymes involved in the maintenance of cellular redox homeostasis, and a number of the genes involved in iron storage and transport (Zheng et al., 2015). The experimental results described above also reveal the involvement of Nrf2 in the regulation of ferroptosis by Shenfu injection, and the status of Akt and GSK-3β as upstream molecular markers of Nrf2 (Zhou et al., 2020). Shenfu injection can reduce the release of myocardial inflammatory factors, inhibit the activation of myocardial NF-кB in septic mice, attenuate septic cardiomyocyte apoptosis, and thus improve cardiac dysfunction by activating the Akt/GSK-3β signaling pathway (Zhou et al., 2024). Our results show that Shenfu injection induced Akt and GSK-3β phosphorylation and Nrf2 expression in a dose-dependent manner, and that the effect of Shenfu injection on the phosphorylation of GSK-3β and Nrf2 expression was inhibited by LY294002—weakening the therapeutic effect of Shenfu injection on cardiac tissue injury and cellular ferroptosis in yang-deficient CHF rats.

In conclusion, this study has demonstrated that Shenfu injection can inhibit ferroptosis in yang-deficient CHF through the activation of the Akt/GSK-3β/Nrf2 pathway, revealing new avenues for the development of clinical novel therapies for the treatment of yang-deficient CHF.

## Figures and Tables

**Table:** Primers.

Genes	Forward	Reverse
Nrf2	ACATTTCAGTCGCTTGCCCT	TTGTGTTCAGCGAAATGCCG
Ptgs2	TGAGTACCGCAAACGCTTCT	TCTAGTCTGGAGTGGGAGGC
SLC7A11	TGCCCGGATCCAGATTTTCC	CAGATTGCAAGGGGGATGGT
GPX4	ACGCCAAAGTCCTAGGAAGC	CTGCGAATTCGTGCATGGAG
SLC3A2	CGAGAGGCATAGCTGGTCTG	CTGCCACTCAGCCAAGTACA
ACSL4	AGCGCAGACCTGCTTTAAGT	TTCCAGCACCGCATGATTCT
GAPDH	GTCGGTGTGAACGGATTTG	TCCCATTCTCAGCCTTGAC

Note: Nrf2, NF-E2-related factor 2; Ptgs2, Cyclooxygenase 2; SLC7A11, solute carrier family 7a member 11; GPX4, glutathione peroxidase 4; SLC3A2, solute carrier family 3 member 2; ACSL4, acyl-CoA synthetase long-chain family member 4; and GAPDH, glyceraldehyde-3-phosphate dehydrogenase.

## Data Availability

The datasets used and/or analyzed in the present study are available from the corresponding author upon reasonable request.
